# Changes in body composition, blood lipid profile, and growth factor hormone in a patient with Prader–willi syndrome during 24 weeks of complex exercise: a single case study

**DOI:** 10.20463/jenb.2018.0006

**Published:** 2018-03-31

**Authors:** Hee Joung Joung, In Soo Lim

**Affiliations:** 1. Department of Physical Education, Seoul National University, Seoul Republic of Korea; 2. Department of Physical Education, Chang Won National University, Chang Won Republic of Korea

**Keywords:** Prader–Willi syndrome (PWS), complex exercise, blood lipid profiles, growth factor hormone

## Abstract

**[Purpose]:**

Prader–Willi syndrome (PWS) is a genetic disorder characterized by excessive appetite with progressive obesity and growth hormone (GH) deficiency. Excessive eating causes progressive obesity with increased risk of morbidities and mortality. Although GH treatment has beneficial effects on patients with PWS, adverse events have occurred during GH treatment. Exercise potentially has a positive effect on obesity management. The purpose of this research was to examine the effects of 24-week complex exercise program on changes in body composition, blood lipid profiles, and growth factor hormone levels in a patient with PWS.

**[Methods]:**

The case study participant was a 23-year-old man with PWS who also had type II diabetes mellitus because of extreme obesity. Complex exercises, including strength and aerobic exercises, were conducted 5 times one week for 60 minutes per session, over 24 weeks. Blood sampling was conducted five times: before and at 8, 16, 20, and 24 weeks after commencement of the exercise program.

**[Results]:**

Weight, fat mass, triglycerides/high-density lipoprotein (TG/HDL) ratio, mean blood glucose, and GH decreased after training. Blood insulin and insulin-like growth factor (IGF-1) levels increased after training. At 15 and 20 weeks, insulin injection was discontinued. Insulin levels increased and average blood glucose decreased to normal levels; IGF-1 increased continuously during the 24-week exercise program.

**[Conclusion]:**

Conclusion] Twenty-four weeks of complex exercises had a positive effect on obesity and diabetes in the patient with PWS. Therefore, long-period complex exercises might be an effective intervention for improvement of metabolic factors in PWS patients.

## INTRODUCTION

Prader–Willi Syndrome (PWS) is a genetic disorder first reported by Prader, Labhart, and Willi in 1956^1^. The exact cause of PWS is yet to be clearly identified; however, it is assumed to be caused by a problem with genomic imprinting in a certain region of chromosome 15 (15q11-q13)^2^. The clinical symptoms of patients with PWS change as they grow older. At infancy, the primary symptom is feeding difficulty and hypothermia. At the age of two or three years, they experience a sudden spike in appetite and bulimia and become obese before the age of six^1^. Patients with PWS show a tendency toward obesity and low height because of a very high body fat percentage but low muscle mass and bone content^3-5^. Extreme obesity causes complications such as arteriosclerosis, hyperlipidemia, cardiovascular diseases, sleep disorder, type II diabetes, etc.^6^. If the patients succeed in controlling obesity, they can live into their fifties; if not, they die early due to complications from obesity^7^. For this reason, preceding studies on patients with PWS highlight that the primary treatment for the disease does not aim at complete recovery but at prevention, thus treating associated secondary diseases early is more important^8^.

For early prevention and treatment, medication is given to patients with PWS as they all have growth hormone (GH) deficiency, and one of the most important causes of the disease is thought to be a problem in the secretion of GH and insulin-like growth factor 1 (IGF-1) due to dysfunction of the hypothalamus^9^. However, after reports of side effects including the progression of scoliosis, saccharometabolic disorders, increase in type II diabetes, tonsillar hypertrophy, and sudden death in patients with PWS treated with GH medication, preceding studies have highlighted the importance of exercise treatment for PWS^10-15^. However, there have been only a few studies on exercise in patients with PWS. One study investigated changes in body composition after aerobic exercise^16^ and another reported increase in total weight without fat after performance of muscle strengthening exercises^17^.

Based on these preceding studies, this paper reports on changes in the body composition, blood lipid profile, and growth factor hormone during a 24-week period of performance of complex exercises in a patient with PWS. As patients with PWS are treated with a wide variety of medicines, a control of the medicine factor is impossible in a clinical test. In addition, many restrictions exist in selecting participants of similar conditions for a test as patients with PWS show a wide range of clinical symptoms. Therefore, this study was designed as a single subject study that analyzed changes in the body composition, blood glucose (insulin, glucose), blood lipid profile (triglycerides (TG), total cholesterol (TC), low-density lipoprotein cholesterol (LDL-C), high-density lipoprotein cholesterol(HDL-C), TG/HDL, TC/HDL) and GH (IGF-1) in stages during the 24-week period of the performance of complex exercises: before the beginning of the exercise and in the 8th, 16th, 20th, and 24th week.

## METHODS

### Development history of the participant

The PWS patient was 23 years old on enrollment. He was born vaginally and weighed 2.3 kg at birth; however, the weight decreased to 2.1 kg because of dyspnea and disturbance of lactation. The baby did not show active movements even past his first birthday. At the ages of 2–3 years, the level of his language development was lower than normal; however, his movements became more active and his weight increased in accordance with an increasing appetite, making his mother believe that the child’s development was just later than that of other children. However, he began to be obsessed with eating from the age of 5 years, and his weight increased significantly after the age of 9 years because of sitomania. He visited the clinic at the age of 15 years for a symptom of extreme obesity and was diagnosed with type II diabetes. Then, PWS was suspected because of the history of the patient’s abnormal development and a clinical distinction of unusually small hands and feet, thus the patient received a chromosome test. After the patient was diagnosed with PWS due to the loss of chromosome 15, the patient’s mother tried to control his weight by controlling his diet; however, it failed as the mother could not stay with the patient all day long because of her work. Weight control eventually ended up in failure, and the weight of the patient increased even further significantly. Subsequently, exercises such as walking, walking on the treadmill, and swimming were tried; however, the patient tired of them easily and always quit halfway. The patient was taking Diabex Tab, Euglex Tab, and Lipitor Tab for diabetes, and Lantus Solosta and Novorephid were administered to him. He measured his blood glucose level four times a day―before breakfast, two hours after lunch, before dinner, and before going to bed―and wrote the levels down on paper to report changes in the blood sugar level to the doctor at every three-monthly visit. The patient displayed grade II intellectual disability.

At the time of the case study enrollment, the patient was working at a career center for people with disabilities located in C city, Seoul. He was 23 years old with 151.1 cm height, 107.0 kg weight, and 51.3% body fat percentage, which made him extremely obese. We explained to the patient and his parents with a summary of this study and completed an informed consent form. After obtaining the participants’ consent and parent’s approval, we collected the data.

### Body composition

The body composition of the patient was measured using the bio-electrical impedance analysis (BIA) method (Inbody 370, Biopsace, Seoul, Korea) to measure the height, weight, skeletal muscle mass, body mass index (BMI), and body fat percentage 12 times, once every two weeks.

### Blood sampling and analysis

For blood sampling, 10 mL of venous blood was obtained from the upper arm of the patient before breakfast at 9:00–9:30 AM. Originally, blood sampling was planned to be conducted on four occasions during the 24-week period: before the commencement of exercise, in the 8th and 16th weeks, and after the end of the period. However, the patient stopped taking Lantus Solosta in the 15th week and Novorephid in the 20th week, thus one more blood sampling was added in the 20th week from the beginning of the exercise program for a total of five blood samples to more thoroughly observe changes occurring due to the change in medication. An enzymatic colorimetric assay was performed for the TC, TG, HDL, and LDL analyses while an enzymatic kinetic assay and electrochemiluminescence immunoassay (ECLIA) (Hirachi, Japan) were performed for the glucose and insulin analyses. For GH and IGF-1, a chemiluminescence immunoassay (CLIA) (Diasorin, USA) method was used. The blood glucose level was measured using the patient’s own blood glucose monitoring device before breakfast, two hours after lunch, before dinner, and before going to bed. The blood sugar level measured before breakfast was used for the analysis in this study. Self-monitoring of blood glucose level is an important factor demonstrating patients’ response to individual treatment or whether the goal has been achieved after treatment. It is a useful method that is utilized as an indicator for preventing low blood sugar and controlling the level of clinical nutritional treatment, exercise treatment, and medication treatment^18^. The average and standard deviation of the values of all the factors obtained from the analysis of the study were calculated using SPSS statistics program (ver. 18, IBM., Armonk, NY, USA).

### Complex exercise program

A program of 60 minutes combined exercises consisting of 5 minutes of warm-up, 50 minutes of main exercise, and 5 minutes of cool-down was performed five times a week for 24 weeks (Table 1). As the subject of the study had grade II intellectual disability and lacked an experience of long-term exercise and concentration, the intensity of the exercise was determined in consultation with a special physical education expert and an exercise prescription expert. Aerobic exercise using a treadmill and resistance exercises using a rowing machine, squat, seat low, bench press, and butterfly machine were performed. After the four-week preliminary examination, the complex exercise program was commenced. It was composed of a preparation phase from the 1st to 8th week, a promotion phase from the 9th to 16th week, and an adaption phase from the 17th to 24th week. During the preparation phase, a low-intensity exercise of walking on a treadmill for 40 minutes (heart rate reserve (HRR) 30–40%) and a 10-minute 2,000-m resistance exercise using a rowing machine were performed. During the promotion phase, a 40-minute medium-intensity walking exercise (HRR 50–60%) on a treadmill and 2–3 sets of 12 1-repetition maximum (1-RM) 50% resistance exercises (squat and seated low) were performed. During the adaptation phase, 15-minute low-intensity aerobic exercise (HRR 30–40%) and 45-minute high-intensity resistance exercise (1RM 70–80%, squat, seated row, butterfly, and bench press) were performed.

**Table 1.** Complex exercise program

Training		Period (week)	Intensity		Contents	Frequency
			HR max	1R M (%)		
warm-up	stretching	1–24			stretching 5 min	5 times/wk
main exercise	treadmill & rowing machine	preparation phase 1–8	30~40%	-	treadmill 40~50 min	
					rowing machine 2,000 m	
	treadmill & weight training	promotion phase 9–16	50~60%	50~60%	treadmill 40~50 min weight training squat, seated row 12 times, 2~3 set	
	weight training & treadmill	adaptation phase 16–24	30~40%	70~80%	weight training squat, seated row, butterfly, bench press 12 times, 4~5 set	
					treadmill 10~15 min	
cool-down	stretching 5 min	1–24			stretching 5 min	

## RESULTS

### Change in body composition

Table 2 shows the changes in the body composition that occurred as a result of the 24-week-long complex exercises. The weight decreased from 106.9 kg before the commencement of exercise program to 102.1 kg in the 8th week from the beginning, 100.9 kg in the 16th week, 100.5 kg in the 20th week, and 98.3 kg in the 24th week. Lean body mass changed from 50.1 kg before the beginning of exercise to 46.3 kg in the 8th week, 46.0 kg in the 16th week, 47.0 kg in the 20th week, and 49.6 kg in the 24th week. Body fat percentage changed from 53.1% before the exercise to 54.6% in the 8th week, 54.4% in the 16th week, 53.3% in the 20th week, and 51.1% in the 24th week. BMI changed from 46.9 kg/m² before commencement of the exercise program to 44.2 kg/mm² in the 8th week, 43.9 kg/mm² in the 16th week, 44.1 kg in the 20th week, and 44.4 kg in the 24th week.

**Table 2.** Results of body composition in a Prader–Willi Syndrome patient.

Training phase	Period (week)	Weight (Kg)	Lean Body Mass (Kg)	Body fat (%)	BMI (kg/m^2^)
Pre	0	106.9	50.1	53.1	46.9
Preparation phase	2	106.4	49.7	53.3	46.7
	4	104	47.3	54.5	45.6
	6	103.7	46.9	54.7	44.9
	8	102.1	46.3	54.6	44.2
Promotion phase	10	101.3	47.4	53.2	43.8
	12	101.8	47.5	53.3	44.1
	14	101.5	46.2	54.5	43.9
	16	100.9	46	54.4	43.9
daptation phase	18	99.4	45.1	54.6	43.3
	20	100.5	47.0	53.3	44.1
	22	101	47.9	52.6	44.3
	24	101.3	49.6	51.1	44.4

### Change in average blood glucose level

**Fig.1.**
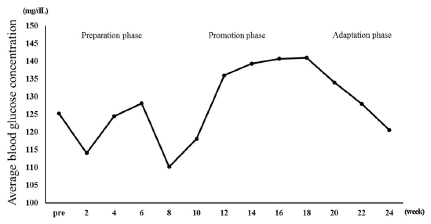
Changes in average blood glucose concentration

The changes in the average blood glucose levels are outlined in Figure 1. During the preparation phase, the average blood glucose levels demonstrated a decreasing trend from 125.19 ± 20.98 mg/dL at the beginning of the exercise program to 110.21 ± 20.98 mg/dL in the 8th week. During the promotion phase, an increasing trend was observed from 136.0 ± 12.72 mg/dL in the 12th week to 140.64 ± 49.67 mg/dL in the 16th week. During the adaptation phase, however, the levels again showed a decreasing trend from 134.0 ± 24.72 mg/dL in the 20th week to 128.0 ± 15.17 mg/dL in the 22nd week and 120.57 ± 26.20 mg/dL in the 24th week.

### Changes in blood lipid component ratio and insulin

The changes in blood lipid profile that occurred in the process of the complex exercise for 24 weeks are presented in Table 3. Insulin levels increased from 2.3 μU/mL before the beginning of the exercise program to 3.8 μU/mL in the 8th week, 8.5 μU/mL in the 16th week, 8.4 μU/mL in the 20th week, and 9.9 μU/mL in the 24th week. Glucose levels increased from 87 mg/dL before the exercise program to 99 mg/dL in the 8th week and 163 mg/dL in the 16th week. Thereafter, it declined to 117 mg/dL in the 20th week and 93 mg/dL in the 24th week. Triglyceride levels changed from 77 mg/dL before the exercise program to 87 mg/dL in the 8th week and then to 76 mg/dL in the 16th week. Thereafter, it increased to 90 mg/dL in the 20th week and decreased again to 77 mg/dL in the 24th week. Total cholesterol changed from 127 mg/dL before the exercise program to 143 mg/dL in the 8th week and to 132 mg/dL in the 16th week. It subsequently increased to 162 mg/dL in the 20th week and to 171 mg/dL in the 24th week. LDH-C increased from 62 mg/dL before the exercise program to 74 mg/dL in the 8th week, 77 mg/dL in the 16th week, 93 mg/dL in the 20th week, and then to 105 mg/dL in the 24th week. HDL-C changed from 41 mg/dL before the exercise program to 56 mg/dL in the 8th week, 43 mg/dL in the 16th week, and to 55 mg/dL in the 20th and 24th weeks.

**Table 3.** Changes in Blood Lipid Profile

Item	pre	8th week	16th week	20th week	24th week
Pre	2.3	3.8	8.5	8.4	9.9
Glucose, mg/dL	87	99	163	117	93
Triglyceride, mg/dL	77	87	76	90	77
Total cholesterol, mg/dL	127	143	132	162	171
LDL cholesterol, mg/dL	62	74	77	93	105
HDL cholesterol, mg/dL	41	56	43	55	55

The changes in the blood lipid component ratio are shown in Figure 1. The TG/HDL ratio changed from 3.0 before the exercise program to 2.5 in the 8th week and 3.0 in the 16th week. Thereafter, it decreased to 1.6 in the 20th week and 1.4 in the 24th week. The TC/HDL ratio decreased from 3.0 before the exercise program to 2.5 in the 8th week, 3.0 in the 16th week, and 2.9 in the 20th week. Thereafter, it increased again to 3.0 in the 24th week.

### Changes in growth hormone

GH increased from 1.07 ng/mL before the exercise program to 4.08 ng/mL in the 8th week and then decreased to 0.41 ng/mL in the 16th week. Thereafter, it demonstrated no significant change at 0.27 ng/mL in the 20th week and 0.41 ng/mL in the 24th week (Figure 3).

**Fig.2.**
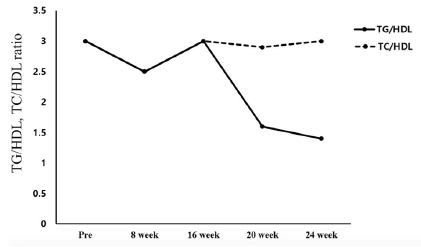
Changes in TG/HDL^1^, TC/HDL^2^ ratio ^1^ Triglyceride (TG) / High-Density lipoprotein Cholesterol (HDL) ratio ^2^ Total Cholesterol (TC) / High-Density lipoprotein Cholesterol (HDL) ratio

**Fig.3.**
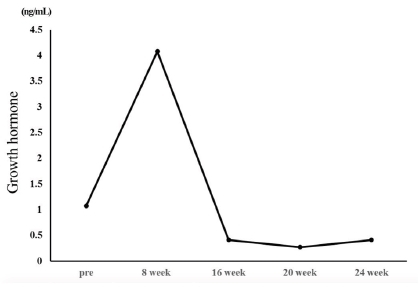
Changes in Growth Hormone levels

**Fig.4.**
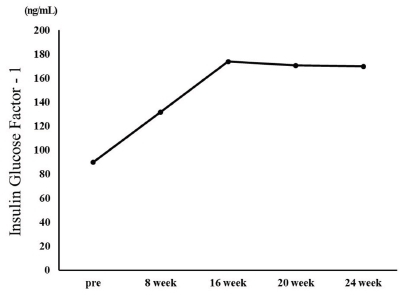
Changes in Insulin-like Growth Factor-1(IGF-1)

IGF-1 increased from 90 ng/mL before the exercise program to 134 ng/mL in the 8th week and 174 ng/mL in the 16th week. Thereafter, it decreased to 170.8 ng/mL in the 20th week and 170.0 ng/mL in the 24th week (Figure 4).

## DISCUSSION

The extreme obesity of patients with PWS increases the incidence of metabolic syndrome such as diabetes and cardiovascular disease and causes early death of patients, thus control of their weight should be the primary factor for treatment^14^. The largest number of studies on weight control in patients with PWS covered clinical GH medication while exercise has been mostly recommended as a natural treatment^19^. Based on the results of preceding research, this study analyzed the changes in the body composition, blood lipid profile, and GH in a PWS patient that occurred during a 24-week-long complex exercise program for improvement of obesity. With respect to body composition, weight (106.9 to 101.3 kg), body fat percentage (53.1 to 51.1%), and BMI (46.9 to 44.4 kg/m2) showed gradual decreases, though slightly. With regard to metabolic syndrome risk factors, the TG/HDL ratio decreased to 1.4 in the 24th week of the exercise program from 3.0 before commencement of the exercise program. The TC/HDL ratio was 3.0 before the commencement of the exercise program and remained at 3.0 in the 24th week. According to Shearman^19^, the lipid component ratio implies risk factors for coronary artery disease and insulin resistance, and the TG/HDL ratio is especially high in patients with insulin resistance. Notably, Attila^20^ stated that the TG/HDL ratio has a positive correlation with obesity and insulin resistance, and insulin resistance increases when the TG/HDL ratio exceeds 3.0. The result of this study suggests that the TG/HDL ratio in the participating PWS patient decreased to below 3.0, lower than the level that creates a risk of insulin resistance and cardiovascular disease, indicating that the 24-weeklong exercise program was effective. The blood insulin concentration increased from 2.3 μU/mL before the exercise program to 9.9 μU/mL after the completion of the exercise program, and the average blood glucose level showed a gradual decrease from 123 mg/mL to 109 mg/mL. According to Burman et al.^21^, Laurance, Brito, and Wilkinson^22^, and Greenswag^23^, patients with PWS experience a severe decline in the insulin level in line with obesity and diabetes, therefore insulin must be constantly administered. Insulin medicines Lantus Solosta and Novorephid were stopped for the participant participant in this study in the 15th and 20th weeks from the commencement of the exercise program, respectively; however, the insulin level increased, and especially, the average blood glucose declined. In an additional test, glycated hemoglobin level declined from 10.9 H before the exercise program to 7.8 H after the exercise program. Considering such a result, it is suggested that the 24-week-long exercise improved insulin sensitivity. Rosenfalck et al.^24^ suggested a correlation between insulin and GH, reporting that insulin resistance is usually observed in GH-deficient patients. In the testing in this study, the GH level of the participant was 1.07 ng/mL before the exercise program and 4.08 ng/mL after eight weeks of exercise. Thereafter, it decreased to 0.41 ng/mL in the 16th week from the beginning of the exercise program and remained at a similar level since then at 0.27 ng/mL in the 20th week and 0.41 ng/mL in the 24th week. In short, the level increased until the eighth week from the beginning of the exercise program; however, it showed a decreasing trend subsequently. GH medicine was administered consistently until before the beginning of the exercise program, and the medication was stopped after the eighth week. Thus, it is implied that the suspension of the GH medication caused the decrease in GH, and the increase in the GH level until the eighth week of exercise was caused by the residual effect of the medication. GH level is assumed to have increased significantly if the exercise was performed along with GH medication. Grugni et al.^25^ reported that patients with PWS with a low GH level also had a low IGF-1 level, and Jin (2007)^26^ reported that IGF-1 level increased as a result of GH medication in patients with PWS. Hoybye^14^ reported that an increase in IGF-1 level from 122 ng/mL to 260 ng/mL was observed as a result of GH medication in adult patients with PWS for six years. However, in this study, IGF-1 increased from 90.0 ng/mL before the exercise program to 170.0 ng/mL after the exercise program even though GH medication for the subject was stopped. Such a result of increase in IGF-1 without GH medication suggests that the effect was due to the exercise.

## CONCLUSION

For this study, complex exercises were performed for 24 weeks by a patient with PWS with dysfunction of the hypothalamus-pituitary gland and GH deficiency, and changes in the patient’s body composition, blood lipid, and GH were analyzed. The weight and body fat decreased after the 24 weeks of exercise while the TC/HDL ratio showed no change. However, the TG/HDL ratio decreased to below 3.0, lower than that which is the level of risk of insulin resistance and cardiovascular disease. Even though the medication of insulin drugs Lantus Solosta and Novorephid were stopped in the 15th and 20th weeks from the commencement of the exercise program, respectively, the blood insulin level of the participant increased, and the average blood glucose level decreased. Blood GH level rose until the eighth week of the exercise and then decreased since then. The rise in the GH level until the eighth week of the exercise is thought to have been caused by the residual effect from the previous medication. The decline in the GH level since the eighth week is thought to have been more greatly affected by the stoppage of the medication than by the exercise. Notably, IGF-1 increased from 90.0 ng/mL before the commencement of the exercise program to 170.0 ng/mL after the completion of the exercise program despite the suspension of the GH medication, suggesting that consistent exercise has an effect of restricting and controlling even a disease known to be incurable.
